# Student perceptions of the education environment in a Spanish medical podiatry school

**DOI:** 10.1186/s13047-018-0252-7

**Published:** 2018-04-10

**Authors:** Patricia Palomo-López, Ricardo Becerro-de-Bengoa-Vallejo, César Calvo-Lobo, Natalia Tovaruela-Carrión, David Rodríguez-Sanz, Marta Elena Losa-Iglesias, Daniel López-López

**Affiliations:** 1 University Center of Plasencia, Universidad de Extremadura, Plasencia, Spain; 2 School of Nursing, Physiotherapy and Podiatry, Universidad Complutense de Madrid, Madrid, Spain; 3 Nursing and Physical Therapy Department, Institute of Biomedicine (IBIOMED), Faculty of Health Sciences, Universidad de León, Ponferrada, León, Spain; 4 Department of Podiatry, University of Seville, Sevilla, Spain; 5 Faculty of Health Sciences, Universidad Rey Juan Carlos, Alcorcón, Spain; 6 Research, Health and Podiatry Unit. Department of Health Sciences, Faculty of Nursing and Podiatry, Universidade da Coruña, Ferrol, Spain

**Keywords:** DREEM, Educational environment, Students’ perception, Podiatry students

## Abstract

**Background:**

The aim of this study was to explore students’ perceptions of the educational environment (EE) in a Spanish school of podiatry. Various aspects of EE were compared by academic year in the program.

**Methods:**

This was a cross-sectional study using a questionnaire to collect perceptions using data from a 2015 survey. Podiatric medical students from Extremadura University participated in this study. EE was assessed with the Dundee Ready Education Environment Measure (DREEM) tool.

The DREEM questionnaire covers five domains of student perceptions, including learning, teachers, academic self-perceptions, atmosphere, and social self-perceptions.

**Results:**

Two hundred thirty-five students participated, resulting in a 90.73% response rate. Participants included similar numbers of students from different years in the program, and most were women. The global EE score was 2.58 out of 4, indicating that students’ perceptions were more positive than negative. Although some weaknesses were detected in this school, students viewed the EE positively in all five DREEM domains. Academic year in the program were generally not related to perceptions of EE.

**Conclusions:**

Podiatric medical students declared, in general, that the EE was more positive than negative in our school, according to the DREEM questionnaire. However, although the results are on the whole good, some areas need to be revised to make improvements.

## Background

The concept of educational environment (EE) is receiving increasing attention due to its impact on the teaching-learning process. Evaluating EE is key to providing high quality education, as has been described in a number of educational studies [1–4]. In 1998 the World Federation for Medical Education highlighted the EE as one of the targets for evaluating medical education [5], and previous studies have shown that educational environments and students’ perceptions of them are associated with academic success, satisfaction with the curriculum and the mode and content of students’ studying [6–8]. EE is a process that allows individuals to collect and evaluate environmental issues, engage in problem solving, and take action to improve the environment, as a result, individuals develop a deeper understanding of environmental issues and have the skills to make informed and responsible decisions [9].

It is not easy to measure and evaluate the EE of a teaching institution because it consists of the sum of students’ individual perceptions of the various items being studied. These perceptions are influenced by a series of individual characteristics such as gender, age and year in school as well as attributes of the degree course itself, such as the educational facilities, teaching staff, course organization and student motivation. A variety of instruments have been used to evaluate EE over the years, such as the Medical School Environment Index (Hutchins, 1961) [10] and others [11, 12]; most of these are currently considered obsolete.

In 1997, Roff et al. [13] at Dundee University developed and validated the Dundee Ready Education Environment Measure (DREEM), which is the tool used in this study. It has since been validated in health science education programs internationally as a method for evaluating the quality of the EE in a wide variety of teaching institutions in countries as far apart as Nigeria, Nepal, India, Greece or Malaysia [1, 9, 14, 15], and it is used in a growing number of research studies around the world [16, 17]. Its use aids in planning improvements by detecting strengths and weaknesses, and it has been widely used to gather data on EE.

The tool was used in this study due to interest in evaluating EE in the Podiatry degree course offered by Extremadura University (Spain), which was created in 1999 and draws its students from various regions of Spain, age brackets and socio-economic classes. Some of the students have already completed other higher education courses. The Faculty of Podiatry is located on the Plasencia Campus of this publicly-funded university, and the 4-year degree course includes annual exams, pre-clinical seminars and practical exams in the University Podiatry Clinic, culminating in a final year dissertation.

Our university constantly strives to offer education of the highest quality, and our study aimed to evaluate the EC as perceived by podiatric medical students at the Plasencia College of Podiatry in Spain. A secondary objective was to assess whether perceptions differed among students by academic year in the program.

## Methods

### Study group

This was a cross-sectional study using a questionnaire to collect perceptions using data collected at the end of the academic year between june 15th and july 15th of 2015 from Freshman, Sophomore, Junior, Senior students of podiatry. Previous studies [2, 4, 6, 9, 18] have revealed variations in the response rate, and for this reason, we administered the DREEM questionnaire to as many students as possible toward the end of the academic year. The test was administered by an assistant professor from nursing department who was not involved with the study nor involved with podiatry student education. Lectures of any podiatry course were not present at any time during the study to avoid possible bias or influence among the students.

After obtaining approval from the university’s Bioethics and Biosafety Committee (Registration number: 46/2015), the DREEM questionnaire was given out to those students who had indicated their willingness to take part in the study. Before starting, participants were given a brief overview of the study’s aims, addressing any doubts they may have had, in particular with regard to the voluntary nature of their participation and the total anonymity of the process.

The DREEM forms were distributed to the students to be filled and returned within 20 min and the students had not permission to talk in order to avoid discussion among them, and those who did not wish to take part or to complete all the sections were free to leave when they wished. Students who could not participate in the study did not have the opportunity to do so to avoid bias due to could have information from their classmates.

Because it was voluntary and anonymous, a separate consent form was collected. The data were handled and stored in accordance with the tenets of the Declaration of Helsinki and to protect confidentiality anonymous data were collected and was not connected to information that can identify the individual participant. Data was also collected regarding the age, gender and academic year of each participant.

### Sample size

It was planned to recruit all the students. The sample size was calculated with software from Unidad de Epidemiología Clínica y Bioestadística, Complexo Hospitalario Universitario de A Coruña, Universidade da Coruña (www.fisterra.com). The calculations were based on the total students of podiatry at Extremadura University (Spain), which amounted to 259 adults on January 1, 2015. It was determined that, based a desired power of 80% with a β level of 20%, and a precision of 3% with an α level of 0.05, with a confidence interval of 95%, for a proportion of 50%, assuming a loss of 15%, at least 134 participants should be included in the study.

### DREEM questionnaire

This instrument contained 50 items evaluated on a 5-point Likert scale. The scoring of items was as follows: 0 = strongly disagree; 1 = disagree; 2 = uncertain; 3 = agree; 4 = strongly agree). Higher overall scores indicate a more positive evaluation of each aspect of the EE.

Our methods follow those of Al-Naggar et al [15] and Chandran et al. [19]. A brief summary of the methods follows. The 50 items were assigned to 5 different domains:

- D1: Students’ perception of learning, consisting of 12 items, with a maximum score of 48.
- D2: Students’ perception of teachers, consisting of 11 items, with a maximum score of 44.
- D3: Students’ academic self-perceptions, consisting of 8 items, with a maximum score of 32.
- D4: Students’ perceptions of atmosphere, consisting of 12 items, with a maximum score of 48.
- D5: Students’ social self-perception, consisting of 7 items, with a maximum score of 28.

Results were tallied for each item and each domain; additionally, an overall score was computed. Nine of the items (4, 8, 9, 17, 25, 35, 39, 40 and 50), are scored in reverse, so were subtracted. Items with a mean score of 3.5 or more were considered “real positive”. The results are presented as percentages of their respective subscales [20, 21].

The mean scores at each level of analysis (i.e., item, domain, and overall) are grouped into four categories (0–50, 51–100, 101–150, and 151–200), each associated with a specific interpretation [22]. The maximum score is 200. The interpretation of the overall scores is as follows: 0–50: very poor; 51–100: a number of problems; 101–150: more positive than negative; and 151–200: excellent.

### Data analysis

All data were explored for normality using the Kolmogorov-Smirnov test, and all variables showed a normally distribution (*P* > 0.05). Descriptive statistical analysis was performed using mean ± SD and median for quantitative variables. The categorical variables were described by frequency and percentage. To compare groups, an independent t-test was used. Chi-square test was used to compare categorical variables. A *p* value < 0.05 with a confidence interval of 95% was considered statistically significant for all tests (SPSS for Windows, version 20.0; SPSS Inc., Chicago, Illinois).

## Results

A total of 259 students enrolled in the Podiatry degree course at Extremadura University (Spain) participated in this study; the majority of them were female. Some students were excluded from analysis because they either failed to complete or return the questionnaire.

A breakdown of the participants by year revealed that 58 first-year students, 64 s-year students, 57 third-year students and 56 fourth-year students completed the questionnaire. This total of 235 participants out of a possible 259 enrolled on the podiatry degree course gives a response rate of 90.73%. Participants’ ages ranged from 18 to 48, the mean age being 22.5 years. In terms of gender, males accounted for 29.8% of the total respondents, and females 70.2%. Slightly over half of the students came from the university’s home region of Extremadura, the rest being from a variety of regions all over Spain, such as Madrid, Andalusia, Cantabria and the Basque Country, among others.

All participants completed the DREEM questionnaire. Table 1 shows sample size, gender and age.

**Table 1:** Socio-demographic characteristics of the podiatric medical students at Extremadura University who participated in this study (*n* = 235)

Year of study	Number participant	Male (%)	Female (%)	Age, Mean(SD), Median
First	58	14 (24.1)	44 (75.9)	21.07 (4.51), 20
Second	64	19 (29.7)	45 (70.3)	22.59 (4.66), 21
Third	57	18 (31.6)	39 (68.4)	22.61 (2.67), 22
Fourth	56	19 (33.9)	37 (66.1)	23.75 (4.18), 23
Total	235	70 (29.8)	165 (70.2)	22,50 (4.18), 22

*Abbreviation*: *SD* standard deviation

The fact that there were more females than males is statistically highly significant (*P* < 0.001, Chi-square test). The distribution of students by year, however, was uniform (*P* = 0.883, Chi-square test). The percentage of female participants decreased over the years, but not significantly (*P* = 0.698, Chi-square test). With regard to age, there was no significant difference between males and females, but there are significant differences between year groups, with age logically increasing from one year to the next (*P* < 0.001).

It is important to note that responses were given for all items by all participants, giving a total of 11,750 responses, with each item receiving scores across the whole of the range (0–4), although not homogeneously. Only 5% of the items received a 0, the most popular score being 3 (37%).

The full score was 129.21 out of a total maximum score of 200, which is more positive than negative. Ninety percent of participants gave overall scores of between 101 and 200 on the Likert scale, which is very positive (Table 2).

**Table 2:** Mean (SD), median and number of student (percentage) included in each category, associated with the interpretation (*n* = 235). The score was 129.21 out of a total maximum score of 200

DREEM	Mean (SD), Median	Number of student (%) included in each category	Interpretation
Educational environment	129.21 (22.83) 134.00	0–50 = 1 (0.4) 51–100 = 25 (10.6) 101–150 = 175 (74.5) 151–200 = 34 (14.5)	More positive than negative
Learning	30.18 (5.99) 31.00	0–12 = 2 (0.9) 13–24 = 35 (14.9) 25–36 = 170 (72.3) 37–48 = 28 (11.9)	More positive than negative learning
Teachers	27.51 (5.47) 28.00	0–11 = 1 (0.4) 12–22 = 38 (16.2) 23–33 = 168 (71.5) 34–44 = 28 (11.9)	More positive than negative
Academics	22.31 (5.08) 23.00	0–8 = 5 (2.1) 9–16 = 20 (8.5) 17–24 = 129 (54.9) 25–32 = 81 (34.5)	More positive, excellent
Athmosphere	30.85 (7.09) 32.00	0–12 = 6 (2.6) 13–24 = 34 (14.5) 25–36 = 148 (63) 37–48 = 47 (20)	More positive than negative
Social life	18.34 (3.99) 19.00	0–7 = 4 (1.7) 8–14 = 33 (14) 15–21 = 148 (63) 22–28 = 50 (21.3)	More positive than negative

*Abbreviation*: *SD* standard deviation

The practical guide authored by McAleer and Roof [8, 22] was used as a reference to interpret the total mean scores.

Each domain was analysed, and overall. All five domains were more positive than negative. Domain D3: Students’ academic self-perception was the most highly rated, and domain D2: Students’ perception of teachers the lowest.

### D1. Students’ perception of learning

In this domain, which contained 12 items, the score ranged from 2.1 to 3.01. A number of aspects could be improved (see Table 3).

**Table 3:** Domain 1. Students’ perceptions of learning among podiatrist medical students (*n* = 235)

Items	Mean	SD	Median
1. I am encouraged to participate in class	2.45	0.95	3.00
7. The teaching is often stimulating	2.52	0.85	3.00
13. The teaching is student centered	2.40	0.92	2.00
16. The teaching helps to develop my competence	3.01	0.82	3.00
20. The teaching is well focused	2.69	0.83	3.00
22. The teaching helps to develop my confidence	2.89	0.88	3.00
24. The teaching time is put to good use	2.18	1.03	2.00
25. The teaching over-emphasizes factual learning	1.73	0.90	2.00
38. I am clear about the learning objectives of the course	2.77	0.95	3.00
44. The teaching encourages me to be an active learner	2.75	0.92	3.00
47. Long term learning is emphasized over short term learning	2.59	0.97	3.00
48. The teaching is too teacher-centered	2.19	1.02	2.00
Total mean score	30.18	5.99	31.00
Maximum score: 48			
Total mean normalized	2.51	0.49	2.58

*Abbreviation*: *SD* standard deviation

Items 16 and 22 received the highest scores, while item 25 received the lowest score. Participants reported that the teaching they receive helps them to develop their professional skills and increases their self-confidence. None of the mean scores reached 3.5 (very positive).

### D2. Students’ perception of teachers

This domain, which included 9 items, was the lowest rated of all. Item 2 had the highest score and item 50 the lowest. The average score for item 2 (3.02) is important; it indicates that students think that “the teachers are knowledgeable” and they come well prepared for class. On the other hand, a high score (2.81) for item 8, “The teachers ridicule the students,” also merits attention.

Item 50: “The students irritate the teachers”, a negative item, scored 1.96; this could mean that this item indicates problem areas and should be investigated closely. The other items scored between 2.00 and 3.00, indicating the aspects of this domain that could be improved (Table 4).

**Table 4:** Domain 2. Students’ perceptions of teachers among podiatric medical students (n = 235)

Items	Mean	SD	Median
2. The teachers are knowledgeable	3.02	0.80	3.00
6. The teachers are patient with students	2.52	0.93	3.00
8. The teachers ridicule the estudents	2.81	1.30	3.00
9. The teachiers are autoritarian	2.20	1.07	2.00
18. The teachers have good communication skills with students	2.65	0.82	3.00
29. The teachers are good providing feedback to students	2.29	0.92	2.00
32. The teachers provide constructive criticism here	2.50	0.91	3.00
37. The teachers give clear examples	2.76	0.94	3.00
39. The teachers get angry in class	2.26	1.18	2.00
40. The teachers are well prepared for their classes	2.54	0.94	3.00
50. The students irritate the teachers	1.96	1.25	2.00
Total mean score	27.5	5.47	28.00
Maximum score: 44	30,1		
Total mean normalized	2.50	0.49	2.54

*Abbreviation*: *SD* standard deviation

### D3. Students’ academic self-perception

Domain D3 was the most highly rated by the students, obtaining a standardised total mean score of 2.78 (Table 5).

**Table 5:** Domain 3. Students’ academic self-perception teachers among podiatrist medical students (*n* = 235)

Items	Mean	SD	Median
5. Learning strategies which worked for me before continue to work for me now	2.80	1.10	3.00
10. I am confident about my passing this year	3.08	1.06	3.00
21. I feel I am being well prepared for my profession	2.95	0.94	3.00
26. Last year’s work has been a good preparation for this year’s work	2.57	1.08	3.00
27. I am able to memorize all I need	2.50	1.05	3.00
31. I have learned a lot about empathy in my profession	2.87	0.96	3.00
41. My problem solving skills are being well developed here	2.47	0.91	3.00
45. Much of what I have to learn seems relevant to a career in healthcare	3.07	1.04	3.00
Total mean score	22.3	5.08	23.00
Maximum score: 32			
Total mean normalized	2.78	0.63	2.87

*Abbreviation*: *SD* standard deviation

In the analysis of the 8 individual items, items 10 and 45 showed the highest scores, although the other 6 items obtained similar scores. The students were very confident that they were going to pass at the end of the year, and much of what the students have to learn seems relevant to a career in healthcare. Their responses to item 21 indicated that they think they are being well prepared. Therefore, although there is always room for improvement, we believe that we are fulfilling our objectives in this domain.

### D4. Students’ perception of atmosphere

This domain included 12 items. The highest mean score was for item 33: “I feel comfortable in class socially”; items 12 and 17 were awarded the lowest scores. We believe that if students feel comfortable socially this will have a positive impact on their learning. Item 12: “This school is well timetabled” scored 1.56, the lowest of all the items, and indicates an area in which there is room for a great deal of improvement. This score could be attributed to the high workload, the enormous diversity of groups for practicals and the wide spread of hours over which lectures are scheduled. Item 17: “Cheating is rampant in this school” obtained a mean score of 1.87, which is also negative and a cause for concern; solutions must be found to prevent this from occurring (Table 6).

**Table 6:** Domain 4. Students’ perceptions of atmosphere among podiatric medical students (*n* = 235)

Items	Mean	SD	Median
11. The environments are relaxing during the clinical teaching	2.89	1.05	3.00
12. This school is well timetabled	1.56	1.28	2.00
17. Cheating is a ramp in this school	1.87	1.37	2.00
23. The atmosphere is relaxing during lectures	2.61	1.01	3.00
30. There are opportunities for me to develop interpersonal skills	2.51	0.86	3.00
33. I feel comfortable in class socially	3.02	1.08	3.00
34. The atmosphere is relaxing during seminars/tutorials	2.83	1.04	3.00
35. I find the experience disappointing	2.60	3.00	3.00
36. I am able to concentrate well	2.61	3.00	3.00
42. The enjoyment outweighs the stress of the course	2.83	1.11	3.00
43. The atmosphere motivates me as a learner	2.70	0.93	3.00
49. I feel able to ask the questions I want	2.83	1.16	3.00
Total mean score	30.8	7.09	32.00
Maximum score: 48			
Total mean normalized	2.57	0.59	2.66

*Abbreviation*: *SD* standard deviation

### D5. Students’ social self-perception

Of the 7 items included in this domain, items 15 and 19 scored the highest, while item 3 scored the lowest (1.69), indicating a problem area (see Table 7). The score of 3.34 awarded to item 19 was the maximum of all the 50 items in the questionnaire, reflecting that students think they have a good social life, good friends in the School of Podiatry and feel comfortable here. The same cannot be said, however, for item 3: “There is a good support system for students who get stressed”. We believe this perception is due to insufficient information, because the university offers a “Student Support Unit” which, according to the statistics, is hardly ever used. Nevertheless, reducing the breadth of the syllabus and introducing more innovative elements could help to reduce student stress levels. This item needs urgent attention.

**Table 7:** Domain 5. Students’ social self-perceptions among podiatric medical students (*n* = 235)

Items	Mean	SD	Median
3. There is a good support system for students who get stressed	1.69	0.92	2.00
4. I am too tired to enjoy the course	2.38	1.19	2.00
14. I am rarely bored on this course	2.00	1.00	2.00
15. I have good friends in this school	3.17	1.06	4.00
19. My social life is good	3.34	1.00	4.00
28. I seldom feel lonely	2.84	1.19	3.00
46. My accommodation is pleasant	2.92	0.94	3.00
Total mean score	18.3	3.99	19.00
Maximum score: 28			
Total mean normalized	2,62	0.57	2.71

*Abbreviation*: *SD* standard deviation

Table 8 shows the DREEM overall and domain mean scores among the podiatric medical students who completed the questionnaire. The overall score was 129/200 (SD 22.83), indicating that the podiatric medical students’ perceptions of the EE of the school were more positive than negative. The total mean score for D1 was 30.1/48 (SD 5.99); for D2, 27.5/44 (SD 5.47); for D3, 22.3/32 (SD 5.08); for D4, 30.8/48 (SD 7.09); and for D5, 18.3/28 (SD 3.99). The students’ perceptions of the EE were positive for all five DREEM domains or subscales.

**Table 8:** DREEM domains for podiatric medical students (*n* = 235)

Domain DREEM	Number of questions	Maximum DREEM score	Mean	SD
Domain 1. Learning	12	48	30.1	5.99
Domain 2. Teachers	11	44	27.5	5.47
Domain 3. Academics	8	32	22.3	5.08
Domain 4. Atmosphere	12	48	30.8	7.09
Domain 5. Social Life	7	28	18.3	3.99
Total DREEM score	50	200	129.0	22.83

*Abbreviation*: *SD* standard deviation

It should also be noted that in this study, the correlation coefficients of standardised domain scores are non-null and highly significant, ranging from 0.306 to 0.702, which indicates that when students have a low perception in one domain they likely maintain this view in all the others; the converse is also true, namely that when their perception of a given domain is high, this also extends to the other domains.

### DREEM scores by academic year

All domains show fairly homogeneous results by year, with the exception of D4, where first-year students have a higher perception than those of other years (see Table 9). Domains D2 and D4 contain the largest number of items with the highest statistical significance. Item 10: “I am confident of passing the examination this year”, in domain D3, received the highest score of all amongst fourth-year students. First-year students gave item 17: “Cheating is rampant in this school” (domain D4) a mean score (SD) of 2.67 (1.40), the most statistically significant of all and very different from the scores for other years.

**Table 9:** The most statistically significant items by year group for the podiatric medical degree course offered on the Plasencia Campus, Extremadura University, Spain (2015) (*n* = 235)

# Domain/Question	First Mean (SD)	Second Mean (SD)	Third Mean (SD)	Fourth Mean (SD)	*P*-value
D1: Students’ perception of learning					
1. I am encouraged to participate in class	2.17 (0.92)	2.38 (1.07)	2.56 (0.84)	2.71 (0.86)	0.014
24. The teaching time is put to good use	2.72 (0.89)	2.08 (1.01)	2.09 (0.71)	1.84 (0.98)	0.001
D2: Students’ perception of teachers					
2. The teachers are knowledgeable	2.91 (0.62)	3.17 (0.96)	2.95 (0.71)	3.02 (0.82	0.027
29. The lecturers are good at providing feedback to students	2.05 (0,84)	2.30 (0.77)	2.32 (0.89)	2.48 (1.12)	0.046
37. The teachers give clear examples	2.74 (0.78)	2.64 (0.96)	2.63 (1.01)	3.04 (0.95)	0.030
39. The teachers get angry in class	2.66 (1.27)	1.97 (1.12)	1.98 (1.02)	2.48 (1.16)	0.001
50. The estudents irritate the teachers	2.28 (1.15)	1.34 (1.18)	2.23 (1.19)	2.07 (1.27)	0.001
D3: Students’ academic self-perception					
10. I am confident of passing the examination	3.07 (0.72)	2.92 (1.15)	3.45 (1.29)	3.45 (1.29)	0.006
31. I have learned a lot about empathy in my profession	2.76 (0.88)	3.06 (0.94)	2.56 (0.94)	3.07 (0.86)	0.002
D4: Students’ perceptions of atmosphere					
11. The atmosphere is relaxed during clinical	3.21 (0.95)	2.81 (1.12)	2.63 (1.17)	2.89 (0.88)	0.015
12. The course timetable is well charted	1.34 (1.08)	0.92 (0.00)	1.89 (1.19)	2.16 (1.26)	0.001
17. Cheating is rampant in this school	2.67 (1.40)	1.52 (1.32)	2.16 (1.33)	1.16 (0.86)	0.001
23. The atmosphere is relaxed during lectures	2.76 (0.86)	2.25 (1.14)	2.63 (1.06)	2.84 (0.84)	0.008
34. The atmosphere is relaxed during seminars/tutorials	3.24 (0.82)	2.66 (1.02)	2.47 (1.13)	2.96 (1.04)	0 .001
D5: Students’ social self-perception					
3. There is good support system for students who get stressed	1.97 (0.70)	1.34 (0.97)	1.81 (0.87)	1.67 (0.99)	0.002
46. My accommodation is pleasant	3.14 (0.80)	3.17 (0.90)	2.63 (1.01)	2.71 (0.92)	0.001

*Abbreviations*: *DREEM* Dundee ready Education Environment Measure, *SD* standard deviation

High scores were also awarded by first-year students to the atmosphere in lectures, seminars and tutorials. This may be due to the fact that they have fewer practicals and suffer less stress than students in other years. However, the differences between scores by year group in this aspect are slight.

Second-year students gave the lowest scores, with those of the other three year groups being broadly similar, although none reached 3.5 (very positive) (Table 10).

**Table 10:** Mean (SD) values of the EC and the domains scores for the sex of podiatric medical students (*n* = 235)

Domain DREEM	Mean (SD), Years.				
	First	Second	Third	Fourth	p-value
Educational environment	2.66 (0.32)	2.52 (0.45)	2.52 (0.50)	2.61 (0.51)	0.237
Learning	30.99 (4.59)	29.79 (6.03)	30.14 (6.29)	28.93 (6.92)	0.782
Teachers	27.98 (4.40)	26.76 (5.47)	27.03 (5.42)	28.39 (6.42)	0.268
Academics	22.32 (3.65)	22.14 (5.30)	21.59 (5.01)	23.21 (6.08)	0.119
Atmosphere	32.75 (5.31)	29.26 (6.96)	30.05 (8.30)	31.51 (7.16)	0.025
Social Life	19.31 (3.09)	18.39 (4.15)	17.64 (4.36)	18.01 (4.13)	0.219

*Abbreviation*: *DREEM* Dundee Ready Education Environment Measure, *SD* standard deviation

The majority of students who participated in this study were female; however, there were no significant differences between scores awarded by men and women (Table 11).

**Table 11:** Mean (SD) values of the EC and the domains scores for the sex of podiatrist medical students(n = 235)

Domain DREEM	Male Mean (SD)	Female Mean (SD)	*P*-value
Educational environment	2.53 (0.48)	2.60 (0.44)	0.270
Learning	29.00 (6.24)	30.68 (5.83)	0.017
Teachers	27.02 (6.50)	27.72 (4.98)	0.695
Academics	21.48 (5.69)	22.66 (4.78)	0.228
Atmosphere	31.14 (6.70)	30.70 (7.27)	0.791
Social Life	18.08 (3.96)	18.46 (4.00)	0.380

*Abbreviation*: *DREEM* Dundee Ready Education Environment Measure, *SD* standard deviation

## Discussion

EE is an important factor in determining the effectiveness and success of a medical school curriculum [3]. Therefore, our study aimed to evaluate the EE as perceived by podiatric medical students at the Plasencia School of Podiatry, Spain. A secondary objective was to determine the different perceptions among students from different years and between sexes. The overall mean DREEM score in our study was 129/200, which fell well inside the range (101–150) indicative of a “more positive than negative” perception of the environment [22].

Although we have been unable to find any studies using the DREEM questionnaire with podiatric medical students in the literature, the tool is widely used around the world, particularly in the context of healthcare education, and above all, according to the literature we have reviewed, in medicine [2, 6, 23]. It has been used in studies of healthcare education [13] to analyse the EE among dental students [9, 18, 19], medical students [2, 3, 6] and medical graduates [10].

Our mean DREEM score of 129/200 was similar to those found in two studies performed in Malaysia and Nepal, in which the mean scores were 125.3/200 [15] and 129/200 [24], repectively Higher overall mean scores were reported in two earlier Malaysian studies, these being 133/200 and 134/200 [25, 26] as well as in Nepal and the United Kingdom (overall scores of 130/200 [1] and 139/200 [27], respectively). Lower overall scores (119/200, 114/200, and 107/200) were reported in multiple studies from India [3, 6]. Lower overall scores were also reported in Sri Lanka, Nigeria, and Trinidad, with overall scores of 108/200 [28], 118/200 [1] and 109/200 [4], respectively. The lowest score, 89/200, was reported in Saudi Arabia at the College of Medicine at King Saud University [29], followed by a score of 97/200 reported by a study of the Canadian Memorial Chiropractic College [30]. The studies reporting higher total scores than we found in our study [17, 24, 27, 31] may indicate that these institutions adopt a more innovative approach to providing a student-centred approach to education [17].

All students perceived “a more positive approach” (30.1/48) regarding their learning; “moving in the right direction” (27.5/44) regarding their teachers; “feeling more on the positive side” (22.3/32) regarding their academic self-perception; “a more positive environment” (30.8/48) regarding the atmosphere; and “not too bad” (18.3/28) regarding their social self-perception. These results should encourage and motivate the curriculum planners in our institute to raise students’ perceptions about their educational environments to the highest level. In our study, the scores for all five subscales showed positive perceptions of the study participants. Nevertheless, there is a need for improvement in all five domains of the EE at the Extremadura University School of Podiatry.

Six DREEM items had scores of 2 or less, suggesting that these items should be examined more closely as they indicate problem areas. The problem areas include an over-emphasis on factual learning (score 1.73), the students’ perception that they irritate their teachers (1.96), dissatisfaction with the scheduling (1.56), concerns regarding cheating (1.87), a lack of support for for students who get stressed 1.69) and a tendency toward boredom (score 2.00). Additionally, the negative statement “The teachers ridicule the students” scored 2.81. These findings indicate that these areas should be examined more closely, as they relate to problem areas. In parallel with our study, a lack of support for students who get stressed was found in a previous study [32]; additionally, in a study conducted in Saudi Arabia [2], this item had a very poor score (0.9).

In our study, seventeen items were identified with means of more than 2.8, and these can be regarded as strengths. They were items 16 (3.01), 22 (2.89), 2 (3.02), 5 (2.80), 10 (3.08), 21 (2.95), 31 (2.87), 45 (3.07), 11 (2.89), 33 (3.02), 34 (2.83), 42 (2.83), 49 (2.83), 15 (3.17), 19 (3.34), 28 (2.84) and 46 (2.92).

Over half of the items in our study scored between 2.00 and 2.80, indicating aspects of the EE that could be enhanced [22]. In domain D1, these items corresponded to student perceptions that their teachers encouraged them to participate in class, the teachers stimulated them to participate in the teaching sessions, the teaching was student-centred, the teaching was well focused and students felt clear about the learning objectives of the course. Scores in this intermediate category in domain D2 suggested that the students felt that their teachers were patient, good at communicating with them, good at providing students with feedback and criticism and well prepared for their classes. In domain D3, areas of improvement for the curriculum were represented by responses to the following questions: “Last year’s work has been a good preparation for this year’s work”, “I am able to memorize all I need” and “My problem solving skills are being well developed here”. Relevant scores in domains 4 and 5 included responses to the following statements: “The environments are relaxing during lectures”, “There are opportunities for me to develop interpersonal skills”, “I feel comfortable in class socially”, “I am able to concentrate well” and “The environments motivate me as a learner”.

Some of the problem areas identified by the study population have also been identified as problems encountered in medical schools with traditional curricula. These problem areas included the perception that teaching is too teacher-centred, authoritarian and fact-oriented [2, 23], and these aspects correlated with increased student fatigue and reduced student enjoyment and performance. Studies have suggested some explanations for this correlation, including an excess of material, lack of guidance regarding priorities for study, and a perception that the students are at risk of superficial learning [33].

We found a high score (2.81) for item 8, “The teachers ridicule the students,” the reason for this score may be that the teachers spend a lot of time in close contact with their students during clinical during clinical rotations, and criticism may be excessively harsh during these sessions. Teachers need to be aware of this and show more respect toward their students.

In our study, there were no significant differences in DREEM scores between males and females, contrary to the findings of a study carried out in Malaysia [15] in which students’ perceptions about their learning environment showed the greatest difference between males and females, with mean scores being two points higher for the latter than for the former. A possible explanation for this finding may be that females perceived factors such as curriculum, structure, focus and goals more positively than males. This is in agreement with a previous study that reported that males and females show different learning styles [34]. However, it is essential to note here that this trend was opposite to the trend found in other studies carried out in the West Indies [4] and Sri Lanka [28], where males gave higher scores than females.

About social section is the domain with a comparatively lower score, and the problems were that there is poor psycology support system for the students who get bored, tired, or stressed during their academic life. There is a serious concern that they are too tired to enjoy their course. The students reported to have good friends and do not feel lonely and have a good social life. Curriculum planners could consider ways to reduce the bulky curriculum and make it more innovative, engaging, and meaningful so as to reduce student boredom and tiredness.

Other studies have reported that gender showed statistically significant variations in the DREEM score. Gender-specific variations in the DREEM score in a study by Al-Hazimi et al. [2] identified that the female students were more satisfied than their male counterparts with the Dundee University Medical School (overall mean DREEM score 139/200).

In our study there were no significant differences in DREEM scores between year of study, but on the other hand Al-Sketty [35], in his study at three institutes of nursing in the Sultanate of Oman, found variations in the DREEM score based on year of study as well as on gender. In another study, this time carried out in a dental college [19], the score was lowest for the fourth year students.

The University of Extremadura has a Psychological Counseling Unit (https://www.unex.es/conoce-la-uex/centros/profesorado/unidad-asesoramiento-psicologico) and in light of the results found an improvement point is to inform students of the existence of this unit and that its use is free for students.

The university also has a teaching guidance service that offers free courses to teachers, so in light of these findings we also encourage teachers to take these courses to improve teaching.

As a limitation of the study, Miles S, et al. (2012) [36] found that DREEM is used in evaluation for diagnostic purposes, and has been used internationally for different purposes and is regarded as a useful tool by users. However, reporting and analysis differs between publications. A variety of non-parametric and parametric statistical methods have been applied, but their use is inconsistent. This lack of uniformity makes comparison between institutions difficult. We agree and there is a need for informed guidelines on its reporting and statistical analysis. Also, a questionnaire can only gather agree or disagree data but not the opinion behind the answer and further research is needed.

## Conclusion

EE affects student learning and development, and a poor EE can hinder the success of even the strongest teachers. Our study is the first to identify perceptions of the learning environment among podiatric medical students in Spain that help us characterize and address the progam’s strengths and weaknesses. In all five domains, students rated the EE more positive than negative.

Key positive findings included that students perceived that the program helps them develop their professional skills and increases their self-confidence. Additionally, they view their teachers as knowledgeable and prepared for their class. Students’ academic self-perception was the highest-rated domain and is one of the strengths identified in this study. Students are very confident that they are going to pass their end-of year examinations and consider that a lot of what they are learning is highly relevant to their professional career. Additionally, the students reported positive attitudes regarding their friends and social life. All these results taken together lead us to feel that we are achieving our set goals; however, there is always room for improvement.

The study also identified a number of negative aspects, namely: “the teaching over-emphasizes factual learning”, “the students irritate the teachers”, “this school is well timetabled” (more negative), “cheating is rampant in this school” and “there is a good support system for students who get stressed”, all of which need to be thoroughly considered in order to improve these areas. Additionally, we need to improve our system for providing specialised support for students suffering from stress and modify the timetable of both lectures and practical exams to adapt them better to students’ needs. These modifications would go a long way toward addressing the deficiencies identified in our school. Furthermore, a change in teachers’ attitudes toward greater empathy with their students, who believe that they irritate their teachers, is needed in order to create a more agreeable learning environment for everyone in the school. Once changes are made, a similar evaluation can be used in the future to assess their efficacy.

## Abbreviations

DREEM: Dundee Ready Education Environment Measure
EE: Educational Environment
SD: Standard deviation.
